# Sex differences in the risk factors of disability among community-dwelling older adults with hypertension: Longitudinal results from the Health, Aging, and Retirement in Thailand study (HART)

**DOI:** 10.3389/fpubh.2023.1177476

**Published:** 2023-06-02

**Authors:** Utoomporn Wongsin, Tuo-Yu Chen

**Affiliations:** Ph.D. Program in Global Health and Health Security, College of Public Health, Taipei Medical University, Taipei, Taiwan

**Keywords:** disability, older adults, adults with hypertension, Southeast Asia, sex differences

## Abstract

**Background:**

Hypertension poses a serious health problem among Thai older adults which could subsequently lead to disability. However, little to no research has been conducted to understand modifiable risk factors of disability among community-dwelling older adults with hypertension in Thailand. In addition, sex is an important social determinant of health, but its role in disability among older adults with hypertension is less clear.

**Objectives:**

This study focused on community-dwelling older adults with hypertension in Thailand and investigated predictors of disability and examined sex differences in the risk factors that were associated with disability in this population.

**Methods:**

Longitudinal data were from the Health, Aging, and Retirement in Thailand (HART) survey (2015–2017; *N* = 916). The outcome variable was difficulty with the activity of daily living at follow-up. Potential risk factors included sociodemographic information, health behaviors/health status, and disability at baseline. Descriptive analysis and logistic regression analysis were employed to analyze the data.

**Results:**

Most of the participants were female and between aged 60 and 69 years old. Being in an older age group (OR = 1.78, 95% CI: 1.07–2.97, *p* < 0.05), having more chronic conditions (OR = 1.38, 95% CI: 1.10–1.73, *p* < 0.01), experiencing obesity (OR = 2.02, 95% CI: 1.11–3.69, *p* < 0.05), and having disability at baseline (OR = 2.42, 95% CI: 1.09–5.37, *p* < 0.05) significantly predicted disability at 2 year follow-up among community-dwelling Thai older adults with hypertension. The effects of these risk factors on disability at follow-up did not differ by sex. However, different risk factors of disability were observed across sexes.

**Conclusion:**

The situations of disability among older adults with hypertension in Thailand are likely to aggravate due to the rapid aging of the population. Our analysis provided useful information regarding significant predictors of disability and sex-specific risk factors of disability. Tailored promotion and prevention programs should be readily available to prevent disability among community-dwelling older adults with hypertension in Thailand.

## Introduction

Disability is often defined as individuals' difficulties performing activities of daily living (ADL) such as bathing, toileting, and feeding as a result of physical or mental health conditions (1–4). The prevalence of such disability among older adults is nearly 38% worldwide, with a higher prevalence in low-income countries (43%) than in high-income countries (30%) (5). Disability in older individuals can have severe consequences, such as reduced physical activity (6) and increased risks of mental health problems (7). Disability also affects family members. Research shows that disability that occurs among family members can lead to financial insecurity in the family and that the cost of taking care of a disabled person increases with the severity of disability (8, 9). Several risk factors of disability among older adults have been identified in previous research. Being older (10–12) and female (10–13) are related to increased odds of disability. Compared to individuals who are married, those who are separated or divorced have higher risks of disability (11–13). Living in rural settings heightens the probability of having disability compared with living in urban settings (11, 12). Socioeconomic status (SES) can also affect the presence of disability. For example, older adults without education are more likely to be disabled compared to those who with some education. Older adults with a lower income level are more likely to be disabled compared to older adults with a higher income level (14). In terms of health behaviors/health status, such as poor vision or hearing (15, 16), poor self-rated health (3, 13, 14), drinking alcoholic beverage (14), having a lower level of physical exercise (12, 14), being overweight (3, 10, 12, 13), losing weight excessively (17), taking five or more medications (3, 13), having a slower gait speed (17, 18), feeling pain (13), having hypertension (6, 19), having at least one chronic condition (11, 12, 14, 15), and being depressed (3, 10, 12, 15) are risk factors of disability among older adults.

Hypertension is one of the most frequently reported chronic conditions among older adults, with over 50% of older adults with this condition globally (20–25). Older adults with hypertension may be more likely to become disabled because hypertension can influence individuals' physical performances [e.g., poor performance on gait speed and balance (26), an accelerated decline of handgrip strength (27), and a poor postural control that increases risks of falling (28)], which can subsequently increase difficulty with performing daily activities. Previous evidence also supports such notion. Studies have shown that hypertension can increase the odds of having a disability, with about one in eight adults with hypertension having disability (20, 21). In addition, evidence also reveals that the chance of developing disability among individuals with hypertension increases with age. For instance, in one study, the researchers found that individuals aged 60 years or above had higher odds of having disability by 1.5 times compared to those aged between 45 and 59 years old (19). Currently, several studies have been conducted to understand disability among older adults with hypertension (19–21). However, research has shown that even at a similar economical level, the prevalence of disability can vary across countries (29). For instance, the estimated prevalence of disability among older adults in low-income countries was 24.9% in Cameroon, 22.4% in Guatemala, 39.2% in India, 9.7% in Nepal, and 29.4% in Maldives (29). Such variance stresses the importance of country-specific investigations.

This study focused on disability among older adults with hypertension in Thailand—a developing country with an upper-middle-income economy. Nearly 19% of Thailand's population are adults aged 60 years or above in 2019, but the proportion of older adults is expected to increase rapidly by 17% from 2019 to 2050 (30). Hypertension has been the most prevalent chronic condition among older adults in Thailand (31). Because advanced age not only increases the chances of having hypertension (32) but also heightens the risks of developing disability among individuals with hypertension (19), a better understanding of factors that are associated with disability among older adults with hypertension in Thailand is needed to provide evidence-based recommendations to the Thai government.

Four studies, to date, have been conducted to understand disability among older adults in Thailand (11, 33–35). However, only two studies find significant relationship between hypertension and disability (11, 35). These studies are limited because they employ a cross-sectional design. In addition, none of them focus on the population of older adults with hypertension. To address this gap, the first goal of this study was to conduct a longitudinal investigation on the risk factors of disability among Thai older adults with hypertension.

Another aspect of disability that remains unclear is the role of sex. Although there is a higher prevalence of self-reported disability [assessed by difficulty with activities of daily living (ADL)] among older females than males (11, 12, 36, 37), several studies have shown no effects of sex on ADL disability (6, 13, 38). Because disability may differ by health conditions (20, 39) and sex (11, 12, 36, 37), a better understanding of sex disparity in disability among older adults with hypertension is warranted. Hence, the second goal of this study was to further examine the relationship between sex and disability and investigate whether risk factors associated with disability differed by sex.

## Materials and methods

### Data

Longitudinal data were from the HART (40), a study that consisted of nationally representative sample of adults aged 45 year old and over. The data are available for academic users from the website of the Center for Aging Society Research (CASR), NIDA, Thailand at http://rc-demo.nida.ac.th/casr/. We used data from 2015 (served as baseline) and 2017 (served as follow-up). Among the original sample (*N* = 5,616) at baseline, 3,718 individuals completed follow-up survey. The final sample size was 916 individuals after excluding individuals who were younger than 60 years old at baseline (*n* = 1,788), did not have hypertension at baseline (*n* = 2,229), and had missing data on key variables in both waves (*n* = 683). Compared to those who were excluded (*n* = 4,700), no significant differences were observed in baseline age (*X*^2^(4, *N* = 916) = 4.91, *p* = 0.297), sex (*X*^2^(1, *N* = 916) = 0.12, *p* = 0.725), and education (*X*^2^(1, *N* = 916) = 1.86, *p* = 0.173) in the current sample.

### Measures

#### Outcome variable: disability at follow-up

During the HART interview, participants were asked whether they had difficulties with the following activities of daily living (ADL) in the past week: Dressing, grooming, bathing, and eating. The responses included “able to do it all by myself,” “need helps sometimes or some steps,” “always need help in some steps,” and “need helps for all steps.” Individuals whose response was “need help sometimes or some steps,” “always need help in some steps,” or “need helps for all steps” were considered as having difficulty. Two additional questions were asked in the survey to assess whether participants experienced incontinence of bladder and bowel. The responses included “yes, all the time,” “yes, more than 15 days a month,” “yes, 5–15 days a month,” “yes, no more than 5 days a month,” and “no.” Individuals whose response was “yes” to incontinence were considered as having difficulty. Disability was conceptualized as any difficulty with performing activities of daily living (ADL). A dummy variable for disability was created with 1 indicating individuals with any difficulty with ADL, otherwise no difficulty (coded as 0).

#### Potential risk factors at baseline

Potential risk factors were selected based on factors that were related to disability. These included *sociodemographic information* [i.e., age (10–12), sex (10–13), education (14), marital status (11–13)], *health behaviors and health status* [i.e., taking hypertension medication (3, 13), chronic conditions (11, 12, 14, 15), self-rated physical health (3, 13, 14), smoking, drinking (14), exercise frequency (12, 14, 17), body mass index (BMI) (3, 10, 12, 13, 17), number of pain locations (13), any hearing impairment (16), any visual impairment (15, 16), and depressive symptoms (3, 10, 12, 15)].

##### Sociodemographic information

Age was assessed by asking participants “*How old are you*?” The responses were categorized into three age groups: 60–69 years old (reference group), 70–79 years old, and 80 years or above. Sex is classified as male (0) and female (1). Education was measured by asking respondents “*What is your highest level of education?*” The responses were from 1 (No formal education) to 8 (Higher than a bachelor's degree). We coded primary school or lower as 0 and secondary school or above as 1. Marital status was coded as married (1) and not married (0).

##### Health behaviors and health status

Taking hypertension medication was assessed by asking “*Are you receiving any treatment or taking any hypertension medication?*” The response was yes/no. We coded 1 for “yes” and 0 for “no.”

Chronic conditions were assessed by asking participants if they had been diagnosed by a doctor with the following chronic conditions: Diabetes, vascular diseases, rheumatism/arthritis, diseases of the bone, kidney diseases, lung diseases, brain cancer, and other cancer. A positive response endorsed a chronic condition. The total number of chronic conditions ranged from 0 to 8.

Self-rated physical health was measured by asking “*In general, how would you rate your physical health status?*” Participants were asked to score on a scale from 1 to 100. A higher score means worse health.

Smoking was measured by asking “*Have you ever smoked cigarettes?*” The responses were “yes, and still smoke now,” “yes, but already quit smoking,” and “never.” We coded 1 for current smokers (yes, and still smoke now) and 0 for not current smokers (“yes, but already quit smoking” or “never”).

Drinking was measured by asking “*Have you ever drunk alcoholic beverages such as liquor, beer or wine*?” The responses were “yes, and still drinking now,” “yes, but already stopped drinking,” and “never.” We coded 1 for current drinkers (yes, and still drinking now) and 0 for not current drinkers (“yes, but already stopped drinking” or “never”).

Exercise frequency was measured by asking “*How often do you exercise?*” The responses were from 1 (7 days a week) to 5 (never). We reversed the coding, such that a higher number indicates more frequent physical exercise.

Body Mass Index (BMI) was calculated by using self-reported weight (in kilograms) divided by squared height (in meters). Individuals were then categorized as underweight (< 18.5 kg/m^2^), normal weight (18.50–24.9 kg/m^2^; reference group), overweight (25.0–29.9 kg/m^2^), or obese (>30 kg/m^2^).

Number of pain locations was assessed by asking individuals to rate their pain on a 4-point scale (no, mild, moderate, and severe pain) in the last month for the following locations: head, shoulder, arms, wrist, fingers, chest, stomach, back, hips, legs, knees, ankles, and toes. Individuals responded mild, moderate, or severe pain were considered as having pain. We summed up all locations for respondents and created a 3-category variable: no pain (reference), one location, and multiple pain location.

Any hearing problems was assessed by asking “Have you ever been diagnosed with the following hearing conditions: hearing loss (one ear), hearing loss (both ears), auditory hallucination, tinnitus, inner ear fluid abnormality, others?” The responses were “yes” and “no.” An endorsement to any of these conditions was considered poor hearing and coded as 1, otherwise 0.

Any visual problems was assessed by asking “Have you ever been diagnosed with the following visual conditions: blind one eye, blind two eyes, myopia, hypermetropia, astigmatism, glaucoma, cataract, pterygium, dry eye/xerophthalmia, others?” An endorsement to any of these conditions was considered poor vision and coded as 1, otherwise 0.

Depressive symptoms were assessed with the 10-item Center for Epidemiologic Studies Depression Scale (41). We followed standardized procedure to calculate scores. Total scores were from 0 to 30.

### Data analysis

Descriptive statistics were employed to describe sample characteristics. Univariate logistic regression analysis was used to investigate the association between each potential risk factor at baseline and disability at follow-up. Significant risk factors found in univariate logistic regression analysis were then entered in multiple logistic regression analysis to investigate baseline independent risk factors of disability at follow-up among community-dwelling Thai older adults with hypertension. To investigate sex disparity in the association of risk factors at baseline with disability at follow-up, we performed moderation analysis and sex-stratified analysis. Regarding moderation analysis, in the multiple logistic regression analysis model, an interaction term between each risk factor and sex was entered one at a time. In sex-stratified analyses, within each sex group, we followed similar steps to identify significant risk factors using univariate logistic regression analysis and then entered them simultaneously in a multiple logistic regression analysis. Stata version 15 (Stata Corp LLC, College Station, TX, USA) was used to perform data. This secondary data analysis was approved by the ethics review board at Taipei Medical University (TMU-JIRB: N202304033).

## Results

Table 1 shows sample characteristics at baseline. Among participants with hypertension at baseline, nearly 4% reported any disability at the baseline, and it was about 14.85% at follow-up. Most participants were female, between aged 60–69 years old, and had an education level of primary school or lower. Nearly 50% of the respondents were married. There were approximately 89% of the participants taking hypertension medication.

**Table 1:** Characteristics of participants.

	**All participants (*N* = 916)**
	**Mean (SD) or** ***N*** **(%)**
Female (yes)	552 (60.26)
Interview method	
Face-to-face interview	916 (100)
Age groups	
60–69 years old	362 (39.52)
70–79 years old	332 (35.15)
80 years old or above	232 (25.33)
Education	
Primary school or lower	858 (93.67)
Secondary school or above	58 (6.33)
**Married (yes)**	455 (49.67)
Taking hypertension medication (yes)	814 (88.96)
Chronic conditions (0–8)	0.59 (0.76)
Number of pain locations	
No pain	263 (28.71)
One location	325 (35.48)
Multiple pain locations	328(35.81)
Depressive symptoms (0–30)	6.42 (3.57)
Self-rated physical health (1–100;	4.17 (1.53)
higher is worse)	
Smoking (yes)	132 (14.41)
Alcohol (yes)	132 (14.19)
Exercise frequency	1.08 (1.37)
Body mass index	
< 18.5 kg/m^2^	98 (10.70)
Between 18.50 and 24.9 kg/m^2^	476 (51.97)
Overweight between 25.0 and 29.9 kg/m^2^	258 (28.17)
Obese > 30 kg/m^2^	84 (9.17)
**Any visual impairment (yes)**	93 (10.15)
Any hearing impairment (yes)	3 (3.28)
ADL difficulty at baseline	
Dressing at baseline (yes)	29 (3.17)
Grooming at baseline (yes)	26 (2.84)
Bathing at baseline (yes)	26 (2.84)
Eating at baseline (yes)	26 (2.84)
Incontinence at baseline (yes)	58 (6.33)
Any disability at baseline (yes)	31 (3.38)
ADL difficulty at follow-up	
Dressing at follow-up (yes)	58 (6.33)
Grooming at follow-up (yes)	42 (4.59)
Bathing at follow-up (yes)	39 (4.26)
Eating at follow-up (yes)	29 (3.17)
Incontinence at follow-up (yes)	93 (10.15)
Any Disability at follow-up (yes)	136 (14.85)

### The association of risk factors at baseline with disability at follow-up

Table 2 provides information from the univariate logistic regression analyses assessing the relationship between risk factors and disability among older adults with hypertension in Thailand. Overall, the results showed that being female, being at an older age group, having more chronic conditions, having poor self-rated physical health, using alcohol, being obese, and experiencing disability at baseline were significantly associated with disability at follow-up. These significant variables were next entered into multiple logistic regression analysis to investigate independent risk factors at baseline of disability at follow-up.

**Table 2:** Univariate logistic regression analysis examining the relationship between baseline risk factors and disability at follow-up among community-dwelling older adults with hypertension in Thailand.

	**Disability at follow-up (*****N*** = **916)**		
	**No disability**	**Disability**	**Odds ratio (95% confidence interval)**
	**Mean (SD) or** ***N*** **(%)**		
Female (yes)	459 (83.15)	93 (16.85)	1.51 (1.03–2.23)^*^
Age groups			
60–69 years old	324 (89.50)	38 (10.5)	ref
70–79 years old	271 (84.16)	51 (15.84)	1.61 (1.02–2.52)^*^
80 years old or above	185 (79.74)	47 (20.26)	2.17 (1.36–3.45)^**^
Education			
Primary school or lower	752 (84.85)	130 (15.15)	ref
Secondary school or above	52 (89.66)	6 (10.34)	0.65 (0.27–1.54)
**Married (yes)**	397 (87.25)	58 (12.75)	0.72 (0.50–1.04)
Taking hypertension medication (yes)	694 (85.26)	120 (14.74)	0.93 (0.53–1.64)
Chronic conditions (0–8)	0.53 (0.71)	0.80 (0.89)	1.50 (1.20–1.85)^**^
Number of pain locations			
No pain	225 (85.55)	38 (14.45)	ref
One location	282 (86.77)	43 (13.23)	0.90 (0.56–1.44)
Multiple pain locations	273 (83.23)	55 (16.77)	1.19 (0.76–1.87)
Depressive symptoms (0–30)	6.47 (3.55)	6.91 (3.81)	1.04 (0.99–1.09)
Self-rated physical health (1–100; higher is worse)	4.14 (1.52)	4.61 (15.72)	1.21 (1.07–1.36)^**^
Smoking (yes)	114 (86.36)	18 (13.64)	0.89 (0.52–1.52)
Drinking (yes)	120 (92.31)	10 (7.69)	0.44 (0.22–0.86)^*^
Exercise frequency	1.08 (1.38)	1.08 (1.47)	1.00 (0.87–1.14)
Body mass index			
< 18.5 kg/m^2^	78 (79.59)	20 (20.41)	1.74 (1.00–3.05)
Between 18.50 and 24.9 kg/m^2^	415 (87.18)	61 (12.82)	ref
Overweight between 25.0 and 29.9 kg/m^2^	223 (86.43)	35 (13.57)	1.07 (0.68–1.67)
Obese > 30 kg/m^2^	64 (76.19)	20 (23.81)	2.12 (1.21–3.76)^**^
**Any visual impairment (yes)**	78 (83.87)	15 (16.13)	1.12 (0.62–2.00)
Any hearing impairment (yes)	27 (90.00)	3 (10.00)	0.63 (0.18–2.10)
Disability at baseline (yes)	19 (61.29)	12 (38.71)	3.88 (1.84–8.18)^**^

^*^The significant value of p < 0.05. ^**^The significant value of p < 0.01.

Table 3 presents the results from the multiple logistic regression analysis. The results showed that being in the age group 80 years old or above (OR = 1.78, 95% CI: 1.07–2.97), having chronic conditions (OR = 1.38, 95% CI: 1.10–1.73), being obese (OR = 2.02, 95% CI: 1.11–3.69), and disability at baseline (OR = 2.42, 95% CI: 1.09–5.37). Specifically, compared to individuals who were between 60 and 69 years old at baseline, those aged 80 or above had increased risks of being disable at follow-up by 78%. Having one additional chronic disease at baseline increased the odds of being disable by 38%. Compared to individuals with normal weight, those with obesity at baseline were twice more likely to be disable later. Compared to individuals without disability at baseline, those who were disabled were approximately 2.5 times more likely to report disability at follow-up.

**Table 3:** Multiple logistic regression analysis investigating independent risk factors at baseline of disability at follow-up among community-dwelling older adults with hypertension in Thailand.

	**All participants (*N* = 916)**
	**Odds ratio (95% confidence interval)**
Female (yes)	1.22 (0.79–1.87)
Age groups	
60–69 years old	ref
70–79 years old	1.48 (0.92–2.37)
80 years old or above	1.78 (1.07–2.97)^*^
Chronic conditions (0–8)	1.38 (1.10–1.73)^**^
Self-rated physical health (1–100; higher	1.13(0.99–1.27)
is worse)	
Drinking (yes)	0.52 (0.25–1.08)
Body mass index	
< 18.5 kg/m^2^	1.50 (0.83–2.68)
Between 18.50 and 24.9 kg/m^2^	ref
Overweight between 25.0 and 29.9 kg/m^2^	1.06 (0.67–1.68)
Obese > 30 kg/m^2^	2.02 (1.11–3.69)^*^
Disability at baseline (yes)	2.42 (1.09–5.37)^*^

^*^The significant value of p < 0.05. ^**^The significant value of p < 0.01.

### The role of sex in the relationship between baseline risk factors and disability at follow-up

The prevalence of disability was 0.98% among males and 2.40% among females at baseline. In moderation analysis, there was no significant interaction terms between sex and risk factors (i.e., age groups, chronic conditions, self-rated physical health, drinking, body mass index, and disability at baseline) at baseline on disability at follow-up (Supplementary Table 1). These results suggest that these factors work in a similar manner between sexes among community-dwelling Thai older adults with hypertension.

Differential risk factors of disability emerged in sex-stratified analyses. Among males, self-rated physical health and disability at baseline were significantly related to disability at follow-up in the univariate logistic analysis (Supplementary Table 2). Only self-rated physical health (OR = 1.37, 95% CI: 1.10–1.69) remained significant in the multiple logistic regression analysis (Table 4). Specifically, having one score higher on self-rated physical health at the baseline was associated with increased risks of being disable at follow-up by 37%. Among females, being in the age group of 70–79 and 80 years old or above, having chronic conditions, being obese, and disability at baseline were significantly related to disability at follow-up in the univariate logistic analysis. In the multiple logistic regression analysis, significant risk factors were being in the age group 80 years old or above (OR = 2.90, 95% CI: 1.55–5.45), having chronic conditions (OR = 1.49, 95% CI: 1.12–1.99), and being obese (OR = 2.49, 95% CI: 1.23–5.05). Specifically, compared to individuals who were between 60 and 69 years old at baseline, those aged 80 or above had increased risks of being disable at follow-up by almost three times. Having one additional chronic disease at baseline increased the odds of being disable by 49%. Being obese at baseline increased the risks of having disability at follow-up by 2.5 times compared to normal weight.

**Table 4:** Stratified analysis examining significant risk factors at baseline associated with disability at follow-up among community-dwelling older adults with hypertension in Thailand.

	**All participants (*****N*** = **916) Odds ratio (95% confidence interval)**	
	**Male**	**Female**
**Age groups**		
60–69 years old		Ref
70–79 years old		1.74 (0.97–3.13)
80 years old or above		2.90 (1.55–5.45)^**^
Chronic conditions (0–8)		1.49 (1.12–1.99)^**^
Self-rated physical health	1.37 (1.10–1.69)^**^	1.02 (0.88–1.19)
(1–100; higher is worse)	
**Body mass index**		
< 18.5 kg/m^2^		1.30 (0.62–2.73)
Between 18.50 and 24.9 kg/m^2^		Ref
Overweight between 25.0 and		1.07 (0.60–1.89)
29.9 kg/m^2^	
Obese > 30 kg/m^2^		2.49 (1.23–5.05)^*^
Disability at baseline (yes)	2.65 (0.59–11.79)	2.11 (0.82–5.41)

^*^The significant value of p < 0.05. ^**^The significant value of p < 0.01.

## Discussion

This study investigated predictors of disability among community-dwelling older adults with hypertension in Thailand. We found that being 80 years old or over, and having more chronic conditions, being obese, and experiencing disability at baseline were significant predictors of disability at 2 year follow-up among community-dwelling older adults with hypertension in Thailand. We also investigated the role of sex in the relationship between risk factors at baseline and disability at follow-up. We did not find any significant interaction effects between sex and risk factors at baseline on disability at follow-up, suggesting that the effects of baseline risk factors on disability at follow-up were similar across sexes. However, significant risk factors at baseline of disability at follow-up were different within each sex group. Poor self-rated health was a significant predictor among older Thai men with hypertension. An advanced age, having more chronic conditions, and being obese were significant predictors among older Thai women with hypertension.

To the best of our knowledge, this is the first study investigating disability among older adults with hypertension in Thailand. Although similar research has been conducted in India (21), Taiwan (20), and China (19), each study has different objectives. For instance, the study from India investigated the prevalence of disability and non-communicable diseases (21), the study from Taiwan investigated the effects of chronic conditions on the experiencing of disability (20), and the study from China examined the relationship between comorbid conditions and the experiences of disability among older adults with hypertension (19). Hence, our study contributes to the literature by demonstrating the predictors of disability among older adults with hypertension.

We found that an older age was a significant predictor of disability in Thai older adults with hypertension. This finding is consistent with previous findings (19–21). One possibility is that advanced age is associated with a higher probability of deterioration of physical health (e.g., poor performance on gait speed and balance), which then limit the performance of ADL. For instance, research has shown that endothelial dysfunction is associated with frailty and sarcopenia (42), and advanced age among individuals with hypertension can worsen already existed endothelial dysfunction (42–44). Another study demonstrated that there is association between poorer cerebral perfusion with slower gait speed among adults with advanced age (45). The underlying mechanisms for older ages and disability among older adults with hypertension could be multidimensional and warrant further investigations.

Previous studies have linked obesity to disability among older adult population (3, 10, 12, 13). Our findings added to the literature by showing that obesity significantly predicted disability among older adults with hypertension. Previous studies have shown that hypertension is the main health condition associated with obesity (46) and that individuals with obesity-related hypertension had a higher prevalence of other diseases (e.g., diabetes mellitus and hyperuricemia) (47) and disability (48) compared to individuals with non-obesity-related hypertension (47). Hence, weight management is crucial for disability prevention among older adults with hypertension.

Being consistent with previous research (11, 12, 14, 15), we found that having one additional chronic condition beyond hypertension increased the odds of having disability at follow-up by 38%. However, the combination of chronic conditions may affect the probability of having disability. For instance, a previous study showed that hypertension existed in most of the combinations of multimorbidity and that the combination of hypertension, depressive symptoms, and arthritis had the strongest association with disability than other combinations among older adults in the United States (49). Such findings suggest specific health conditions co-exist with hypertension may have higher chances to result in disability among older adults. More investigations on the association of the combination of hypertension and other chronic conditions with disability among older adults in Thailand are warranted.

We found that nearly 15% of the respondents had disability at baseline, and such experience determined the experience of disability at 2-year follow-up. Although most disability may recover within the following 6 months of the onset, a significant proportion recovers in the subsequent 2 years (50). Nevertheless, one study found that baseline disability remained to be a significant predictor of disability at 3 year follow-up (6). Such discrepancy in time lag and how disability recovers need further investigation to help identify a better timing for the provision of intervention on disability among individuals with hypertension.

Our findings have practical implications. This study provides information regarding predictors of disability among community-dwelling Thai older adults with hypertension across sexes and within each sex group. Such information can be used by government and agencies to develop programs aiming to delay disability among community-dwelling older adults with hypertension in Thailand. Preventive programs (e.g., home and environment modifications for individuals who are already disable) targeting the high-risk groups (i.e., 80 years or above) are important. Continued efforts to deter the development of additional chronic disease, manage obesity, and improve disability may also help prevent disability among older adults with hypertension. Furthermore, given the existence of sex disparity in the risk factors of disability, program planners should also take these sex-specific factors into account to develop preventive strategies.

## Strengths and limitations of this study

The strength of this study was the use of longitudinal data from a nationally representative sample of Thai older adults. There are limitations in this study. First, we analyzed self-reported data from the HART, which may be affected by common measure bias. Second, the HART only focuses on ADL. Hence, we are unable to estimate the prevalence of difficulty with instrumental activities of daily living and investigate their related risk factors. Last, the data are from Thai older adults with hypertension. The findings can be only generalized to this segment of population.

## Conclusion

The prevalence of disability among community-dwelling older adults with hypertension is expected to soar due to a rapidly aging population in Thailand. Our study provides the first glance of disability among community-dwelling older adults with hypertension in Thailand. The main findings are as follows: (1) The oldest aged group (80 years old and above), chronic conditions, disability at baseline, and obesity are the leading risk factors at baseline associated with disability at 2 year follow-up among older adults with hypertension in Thailand, (2) the effects of risk factors at baseline on disability at follow-up are similar across sexes, and (3) an advanced age, having more chronic conditions, and being obese were significant predictors among older Thai women with hypertension, while poor self-rated health and having disability at baseline associated with disability for older Thai men with hypertension. Therefore, customized promotion and prevention programs (i.e., healthy diet, health behavior modification to prevent obesity and chronic conditions) to promote independence among older adults with hypertension in Thailand are a pressing issue that needs to be addressed and organized to provide effective services for this targeted population.

## Data availability statement

Publicly available datasets were analyzed in this study. This data can be found here: Center for Aging Society Research (CASR), The National Institute of Development Administration (NIDA), Thailand (http://rc-demo.nida.ac.th/casr/).

## Ethics statement

The studies involving human participants were reviewed and approved by the Ethical Review Board at Taipei Medical University. Written informed consent for participation was not required for this study in accordance with the national legislation and the institutional requirements.

## Author contributions

UW and T-YC: conceptualization, methodology, and writing—reviewing and editing. UW: data curation, formal analysis, and writing—original draft. T-YC: investigation and supervision. Both authors contributed to the article and approved the submitted version.
