# Supplementary material for: Sex differences in the risk factors of disability among community-dwelling older adults with hypertension: Longitudinal results from the Health, Aging, and Retirement in Thailand study (HART)
Source: Front Public Health. 2023 Jun 2;11:1177476. doi: 10.3389/fpubh.2023.1177476 (PMC10286628; doi:10.3389/fpubh.2023.1177476)
Supplement: Supplementary file 1 [file Data_Sheet_1.docx]

Supplementary Material

Sex differences in the risk factors of disability among community-dwelling older adults with hypertension: Longitudinal results from the Health, Aging, and Retirement in Thailand study (HART)

Utoomporn Wongsin and Tuo-Yu Chen, PhD.*

* Correspondence: Tuo-Yu Chen: email: otfish@gmail.com

Supplementary tables

Table 1. An interaction term between each risk factor and sex

Table 2. Sex-stratified univariate logistic regression

Supplementary Table 1. An interaction term between each risk factor and sex

|  | All  participants (N=916)  Odds Ratio (95% Confidence Interval) | | All participants (N=916)  Odds Ratio (95% Confidence Interval) | All participants (N=916)  Odds Ratio (95% Confidence Interval) | All participants (N=916)  Odds Ratio (95% Confidence Interval) | All participants (N=916)  Odds Ratio (95% Confidence Interval) | All participants (N=916)  Odds Ratio (95% Confidence Interval) | All participants (N=916)  Odds Ratio (95% Confidence Interval) |  |  |  |  |
| --- | --- | --- | --- | --- | --- | --- | --- | --- | --- | --- | --- | --- |
| Female (Yes) | 1.22  (0.78-1.87) | |  |  |  |  |  |  |  |  |  |  |
| Age groups |  | |  |  |  |  |  |  |  |  |  |  |
| 60-69 years old | ref | | ref | ref | ref | ref | ref | ref |  |  |  |  |
| 70 -79 years old | 1.48  (0.92-2.37) | | 1.24  (0.57-2.69) | 1.48  (0.93-2.37) | 1.48  (0.93-2.38) | 1.48  (0.92-2.37) | 1.50  (0.93-2.40) | 1.48  (0.93-2.40) |  |  |  |  |
| 80 years old or above | 1.78  (1.07-2.97)* | | 0.94  (0.40-2.24) | 1.79  (1.07-2.99)* | 1.79  (1.08-3.00)* | 1.78  (1.07-2.97)* | 1.81  (1.08-3.03)* | 1.78  (1.07-2.98)* |  |  |  |  |
| Chronic conditions (0-8) | 1.38  (1.10-1.73)** | | 1.40  (1.11-1.76)** | 1.23  (0.88-1.70) | 1.38  (1.10-1.73)** | 1.39  (1.10-1.73)* | 1.38  (1.11-1.74)** | 1.38  (1.11-1.73)** |  |  |  |  |
| Self-rated physical health (0-100) | 1.15  (0.98-1.34) | | 0.99  (0.98-1.00) | 0.99  (0.98-1.00) | 0.99  (0.97-1.00) | 0.99  (0.98-1.00) | 0.99  (0.98-1.00) | 0.99  (0.98-1.00) |  |  |  |  |
| Drinking (yes) | 0.52  (0.25-1.08) | | 0.50  (0.24-1.04) | 0.51  (0.25-1.03) | 0.54  (0.26-1.13) | 0.53  (0.24-1.54) | 0.53  (0.26-1.11) | 0.52  (0.25-1.08) |  |  |  |  |
| Body Mass Index |  | |  |  |  |  |  |  |  |  |  |  |
| <18.5 kg/m^2^ | 1.50  (0.83-2.68) | | 1.52  (0.85-2.75) | 1.48  (0.83-2.66) | 1.49  (0.83-2.67) | 1.50  (0.84-2.68) | 1.79  (0.71-4.49) | 1.49  (0.83-2.68) |  |  |  |  |
| Between 18.50 and 24.9 kg/m^2^ | ref | | ref | ref | ref | ref | ref | ref |  |  |  |  |
| Overweight between 25.0 and 29.9 kg/m^2^ | 1.06  (0.67-1.68) | | 1.07  (0.68-1.71) | 1.05  (0.66-1.66) | 1.05  (0.66-1.67) | 1.06  (0.67-1.68) | 1.12  (0.50-2.51) | 1.05  (0.67-1.68) |  |  |  |  |
| Obese >30 kg/m^2^ | 2.02  (1.11-3.69)* | | 2.14  (1.16-3.93)* | 2.01  (1.10-3.68)* | 2.00  (1.09-3.65)* | 2.02  (1.10-3.69)* | 1.37  (0.36-5.17) | 2.02  (1.11-3.69)* |  |  |  |  |
| Disability at baseline (yes) | 2.42  (1.09-5.37)* | | 2.32  (1.04-5.16)* | 2.39  (1.08-5.31)* | 2.40  (1.08-5.32)* | 2.42  (1.09-5.37)* | 2.36  (1.06-5.25)* | 2.53  (0.58-11.03) |  |  |  |  |
| **Interaction terms** |  | |  |  |  |  |  |  |  |  |  |  |
| Female*60-69 years old |  | | 0.80  (0.38-1.69) |  |  |  |  |  |  |  |  |  |
| Female*70-79 years old |  | | 1.03  (0.53-2.00) |  |  |  |  |  |  |  |  |  |
| Female*80 years old or above |  | | 2.09  (0.99-4.41) |  |  |  |  |  |  |  |  |  |
| Female*Chronic conditions |  | |  | 1.22  (0.84-1.77) |  |  |  |  |  |  |  |  |
| Female*Self-rated physical health |  | |  |  | 1.00  (1.00-1.01) |  |  |  |  |  |  |  |
| Female*Alcohol |  | |  |  |  | 1.11  (0.13-9.63) |  |  |  |  |  |  |
| Female*Body Mass Index |  | |  |  |  |  |  |  |  |  |  |  |
| <18.5 kg/m^2^ |  | |  |  |  |  | 1.24  (0.69-2.24) |  |  |  |  |  |
| Between 18.50 and 24.9 kg/m^2^ |  | |  |  |  |  | 0.93  (0.33-2.62) |  |  |  |  |  |
| Overweight between 25.0 and 29.9 kg/m^2^ |  | |  |  |  |  | 1.15  (0.51-2.62) |  |  |  |  |  |
| Obese >30 kg/m^2^ |  | |  |  |  |  | 2.01  (0.50-8.14) |  |  |  |  |  |
| Female*Disability at baseline |  | |  |  |  |  |  | 1.15  (0.22-6.08) |  |  |  |  |
| Note. |  | |  |  |  |  |  |  |  |  |  |  |
| The significant value of p<0.05 (*) | | |  |  |  |  |  |  |  |  | |  |
| The significant value of p<0.01 (**) | | |  |  |  |  |  |  |  |  | |  |
|  | |  | |  | |  |  |  |  |  |  |  |

Supplementary Table 2. Sex-stratified univariate logistic regression

|  | | All participants (N=916) | | |
| --- | --- | --- | --- | --- |
| **Variables** | | Odds Ratio (95% Confidence Interval) | | |
|  | | **Male** | | **Female** |
| Age groups | |  | |  |
| 60-69 years old | | ref | | ref |
| 70-79 years old | | 1.43 (0.67-3.05) | | 1.74 (0.99-3.04)* |
| 80 years old or above | | 1.30 (0.57-3.00) | | 2.86 (1.62-5.03)** |
| Education | |  | |  |
| Primary school or lower | | ref | | ref |
| Secondary school or above | | 0.87 (0.29-2.57) | | 0.54 (0.12-2.36) |
| Married (yes) | | 0.97 (0.48-1.94) | | 0.73 (0.45-1.17) |
| Taking hypertension medication (yes) | | 1.12 (0.38-3.32) | | 0.88 (0.45-1.72) |
| Chronic conditions (0-8) | | 1.32 (0.92-1.90) | | 1.59 (1.21-2.09)** |
| Number of Pain locations | |  | |  |
| No pain | | ref | | ref |
| One location | | 0.48 (0.21-1.10) | | 1.23 (0.68-2.23) |
| Multiple pain locations | | 0.90 (0.43-1.87) | | 1.38 (0.78-2.47) |
| Depressive symptoms (0-30) | | 1.05 (0.96-1.14) | | 1.04 (0.98-1.10) |
| Self-rated physical health (0-100) | | 1.40 (1.13-1.73)** | | 1.12 (0.97-1.29) |
| Smoking (yes) | | 1.29 (0.67-2.48) | | 0.82 (0.10-6.90) |
| Drinking (yes) | | 0.54 (0.25-1.16) | | 0.35 (0.04-2.66) |
| Exercise frequency | | 0.96 (0.75-1.23) | | 1.01 (0.87-1.19) |
| Body Mass Index | |  | |  |
| <18.5 kg/m^2^ | | 2.17 (0.89-5.29) | | 1.50 (0.73-3.08) |
| Between 18.50 and 24.9 kg/m^2^ | | ref | | ref |
| Overweight between 25.0 and 29.9 kg/m^2^ | | 1.10 (0.50-2.43) | | 1.00 (0.58-1.72) |
| Obese >30 kg/m^2^ | | 1.37 (0.38-5.01) | | 2.16 (1.13-4.17)* |
| Any visual impairment (yes) | | 1.08 (0.40-2.91) | | 1.16 (0.56-2.41) |
| Any hearing impairment (yes) | | 0.43 (0.06-3.28) | | 0.99 (0.21-4.58) |
| Disability at baseline (yes) | | 3.94 (0.95-16.36)* | | 3.68 (1.52-8.87)** |
| Note. |  |  |  |  |
| The significant value of p<0.05 (*) | | |  |  |
| The significant value of p<0.01 (**) | | |  |  |
